# Motivation, Academic Anxiety, Depression and Well-being among Doctoral Students Over 50

**DOI:** 10.1093/geroni/igaf122.2506

**Published:** 2025-12-31

**Authors:** Michelle Ackerman, Bettina Shapira

**Affiliations:** National University, Sioux Falls, South Dakota, United States; National University, San Diego, California, United States

## Abstract

Increased accessibility to doctoral programs has resulted in a growing trend of doctoral recipients over the age of 45 (6.9% in 2020; NCSES, 2020). Recent studies suggest doctoral students are at significantly higher risk of negative mental health outcomes such as anxiety and depression compared to the general population and current employees with high levels of education (Leveque et al., 2017; Evens et al., 2018). Older doctoral students may experience greater loneliness, more doubt in their academic abilities, have more difficulty sustaining motivation (Usher & McCormack, 2021) and may have different motivations than traditional-age students (Stehlik, 2011; Templeton, 2021). However, while there is a body of research investigating the association of age with doctoral student outcomes, few previous studies have investigated doctoral students who enrolled age 50+. Social media recruitment yielded 303 participants for this quantitative correlational study. Qualtrics online surveys included demographic questions, questions about motivation to enroll, Academic Anxiety Scale, Patient Health Questionnaire-2, Generalized Anxiety Disorder 2-item, and WHO-5 Well-Being Index. The highest reported motivation for enrolling was interest in the subject matter and love of learning (23.2%), followed by pursuing an academic career (17.9%), and to upgrade, improve or expand employment-related knowledge/ qualifications/skills (16.2%). Age, gender, number of children in the home, household income, course type and university type were associated with mental health outcomes. Discussion focuses on supporting older doctoral students to promote success and well-being. Conclusions are relevant for current and potential older adult doctoral students as well as administrators and faculty of doctoral programs.

